# Living in an invaded existence: a phenomenological lifeworld study of young women’s experiences of peer-perpetrated sexual violence in Sweden

**DOI:** 10.1080/17482631.2025.2530749

**Published:** 2025-07-08

**Authors:** Jenni Isaksson, Maria Lundvall, Lina Palmér

**Affiliations:** Faculty of Caring Science, Work Life and Social Welfare, Department of Caring Sciences, University of Borås, Borås, Sweden

**Keywords:** Caring science, reflective lifeworld research, phenomenology, young women, peer-perpetrated sexual violence, existential caring

## Abstract

**Purpose:**

In Sweden, one in four young women has experienced peer-perpetrated sexual violence. This kind of violence causes significant suffering, negatively impacting health and well-being. This study explores young women’s lived experiences of peer-perpetrated sexual violence, aiming to deepen the understanding of how recovery and well-being can be supported.

**Methods:**

This phenomenological study is based on a reflective lifeworld research approach. Lifeworld interviews with 12 young women (17–25 years old) with a lived experiences of peer-perpetrated sexual violence were conducted.

**Results:**

The essential meaning of the phenomenon is described as “living in an invaded existence where an existential void arises and where the movement of life is disrupted.” This is further described as: existing in a body constantly under attack, viewing the body as an object, fighting an inner battle—a struggle for survival, silence as armour, and navigating a dangerous world.

**Conclusions:**

Sexual violence disrupts life, creates existential wounds, alters young women, and reshapes their views of themselves and the world. For health care professionals, it is important to apply an existential caring approach, in which existential awareness is central to the ability to encounter these women in a unique and meaningful way.

## Introduction

The World Health Organisation (WHO, 2022b) defines sexual violence as any sexual act, attempt to obtain a sexual act, or other acts directed against a person’s sexuality through coercion. Worldwide, 120 million girls and young women under the age of 20 have been subjected to some form of sexual violence (WHO, 2022a). Sexual violence is a human rights violation and a global health crisis of pandemic proportions (WHO, 2022b). In Sweden, 22.4% of young women between the ages of 16 and 19, and 29.2% of women between ages 20 and 24 self-reported experience of sexual violence in 2022. In comparison, among young men of the same age groups, 16–19 years and 20–24 years, 2.1% and 4.4%, respectively, reported experiencing sexual violence during the same period (The National Council for Crime Prevention, 2023). This study focuses on young women’s experiences of peer-perpetrated sexual violence where the perpetrator is of the same age and is someone known to them which includes partners, friends and acquaintances. Most commonly young women between ages 16 and 24 report being subjected to sexual violence by a peer male who was identified as a partner (The National Council for Crime Prevention, 2023; WHO, 2022b) but young women are also being subjected to sexual violence where the perpetrator is a friend or an acquaintance regardless of gender (The National Council for Crime Prevention, 2023).

Transitioning into adulthood is a vulnerable time (Reissing et al., 2012), and a negative first sexual experience constitutes a life stressor that may have long-term impacts on physical and emotional wellbeing (Liu et al., 2022). The transition to adulthood is also a period in which sexual violence victimization is high (Korkmaz et al., 2022; Palm et al., 2016). There are indications that young adults with experience of sexual violence where the perpetrator is someone known to them do not seek health care for help and support for distress related to abuse (Ameral et al., 2020; Zinzow et al., 2022). One explanation could be that they rely on friends for support and help, when needed (Fernet et al., 2022). Another explanation may be that young adults often feel that they are judged and not listened to by healthcare professionals (Ellinghaus et al., 2021; Fehler-Cabral & Ampbell, 2013). Yet another reason for not disclosing sexual violence could also be that young adults do not recognize themselves as victims of violence since the idea that “real rapes” are perpetrated by strangers (Tarzia, 2021). The myths of what a rape is can be so deeply internalized that the individual who has been subjected to sexual violence assumes a personal responsibility for the assault which prevents a disclosure of the violence (Harsey et al., 2017; Russell & Hand, 2017). The stigmatization of sexuality and sexual violence can further complicate the disclosure since it evokes feelings of shame and self-blame (dos Reis et al., 2017; Tyson, 2019).

Additionally, healthcare professionals have commented on how they lack the knowledge and skills needed to address sexual violence and violence against women in general (Lau et al., 2023; Öhman et al., 2024; Tarzia et al., 2021). It is important that sexual violence is identified in a supportive way since unsupportive reactions can lead to more distress and to avoidant coping strategies that may increase the risk of ongoing violence and victimization and delay adequate healthcare (Stockman et al., 2023). It is also crucial to establish trusting and supportive relationships to encourage young adults to disclose their traumatic experiences (Ellinghaus et al., 2021; Stockman et al., 2023).

Sexual violence should be treated as a public health problem due to the magnitude of the problem and because of the negative impact sexual violence has on health (Basile, 2003). Previous research indicates that sexual violence causes long-term suffering like psychological problems, suicidality, substance use and negative sexual health behaviours among young adults (Basile et al., 2020; Palm et al., 2016; Stockman et al., 2023; WHO, 2020). However, the essence of what it means to be a young woman in Sweden who has been subjected to peer-perpetrated sexual violence remains under-researched.

From a caring science perspective, sexual violence can be understood as a kind of suffering that negatively influence health and wellbeing. Caring science explores the human relationship to health, suffering, and caring, as well as it focuses on the existential aspects of human existence (Dahlberg & Segesten, 2010). The core of caring is to strengthen health and wellbeing and can be understood such as experiencing well-being and having the opportunity to carry out both one’s major and minor life projects (Dahlberg, 2011). When developing caring practices aimed at strengthening health and wellbeing, it is significant to gain a deeper understanding of the first-person experience (Carel, 2016). Therefore, the aim of this study is to describe young women’s lived experiences of peer-perpetrated sexual violence.

## Approach and methods

This phenomenological study is based on a reflective lifeworld research (RLR) approach described by Dahlberg et al. (2008). RLR takes its epistemological foundation in the phenomenological and hermeneutical philosophy described by Husserl (1936/1970), Merleau-Ponty (1962/2002), and Gadamer (2013). RLR offers an epistemological and methodological approach to uncovering and understanding lived experiences and meanings. RLR is phenomenon-oriented, meaning that the phenomenon is kept in focus during the entire research process. In phenomenological research, a phenomenon is understood as something as it is experienced by someone. The phenomenon in this study is peer-perpetrated sexual violence, as it is experienced by young women in Sweden.

The epistemological foundation for RLR provides the researcher with methodological principles including openness, flexibility, and bridling (Dahlberg et al., 2008). For the researcher to perceive the various dimensions of the phenomenon, it is crucial to continually reflect on and bridle one’s pre-understandings—personal beliefs, theories, and assumptions that may influence the understanding of meaning and prevent the phenomenon from fully revealing itself (Dahlberg et al., 2008). Openness, flexibility, and bridling contribute to avoid making the indefinite definite by being observant, attentive, and sensitive to the experience described. The world of experience should be allowed to present itself in all its complexity, and by remaining flexible to the phenomenon, this can be made possible (Dahlberg et al., 2008). Openness involves actively seeking new insights during the analysis, remaining open to the phenomenon revealing itself in unexpected ways, and continually questioning and reflecting on both what is already known and what remains undiscovered (Dahlberg et al., 2008). Thus, some degree of pre-understanding is necessary; otherwise, one may not recognize when the phenomenon presents itself in a new way. These principles have been used throughout the research process and can be described as a process in which one’s understanding is actively held within a reflective phenomenological attitude towards the phenomenon in question (Dahlberg, 2006). In this way, the process of understanding is allowed to unfold naturally, without making too-quick decisions about the phenomenon, which can instead show itself to the researcher, and the essential meaning of the phenomena can then be illuminated. This requires a reflective phenomenological attitude to manage the complexities of lived experiences in an open and sensitive manner (Dahlberg et al., 2008).

## Participants and data collection

Twelve women with lived experiences of being subjected to peer-perpetrated sexual violence participated in individual lifeworld interviews (Dahlberg et al., 2008) during 2023. For the purposes of this study, sexual violence had to be perpetuated by a peer, friend, acquaintance or partner, for it not to be mistaken for sexual abuse perpetrated by adults against children. Young women were recruited in Sweden through posts in social media channels for different Swedish organizations that highlight violence in adolescent relationships, and with posters and pamphlets in two universities on the west side of Sweden. The inclusion criteria were that the participants: (1) identified themselves as women, (2) were between 16 and 25 years old, (3) had experienced sexual violence, and (4) the perpetrator(s) was a person in the same age range. Fourteen women showed an interest in participating in the study. The women included in the study were all native Swedes between 17 and 25 years old when the interviews were held, three of whom had children-, and came from diverse geographic origins, such as large cities, suburbs, and rural areas across Sweden. The average age of the first lived experience of sexual violence was 14 years. The majority of perpetrators were young men; however, young women were also perpetrators of violence. The lived experience of sexual violence ranges from a single isolated event to a recurrent assault over the course of several years. Eleven lifeworld interviews were conducted through encrypted zoom channels, and one interview was conducted via telephone. This gave the participants the opportunity to choose a neutral and safe environment during the interview.

Lifeworld interviews (Dahlberg et al., 2008) focusing on lived experience and meanings of the phenomenon of being subjected to peer-perpetrated sexual violence as young women were performed. The methodological principles of openness, flexibility, and bridling guided the interviews and enabled a deeper reflection on the phenomenon. The interviews began with the participants being invited to talk about the phenomenon in an open and reflective manner. An open question that directs the phenomenon was asked: “Can you tell me about that time when you were subjected to sexual violence?”. During the interview, the researcher strived to remain open and pliable towards the phenomenon, as well as bridle the understanding of it by asking participants to describe their experience in all its nuances. Reflective follow up questions were included to deepen the understanding of this phenomenon. Questions like “Can you tell me more about that?”, “How did you feel about that?”, “What does that mean to you?”, “What happened to you then?” The interviews were transcribed verbatim by the interviewer. The average length of the interviews was 83 minutes.

## Data analysis

A phenomenological analysis was conducted according to the methodological principles of RLR, openness, flexibility, and bridling towards the lived experiences and meanings of the phenomenon of being subjected to sexual violence as a young woman (Dahlberg et al., 2008). The process of analysis is described as a movement between the whole (all interviews), parts (meanings), and a new whole (the essential meaning). This includes a movement between closeness and distance towards the lived experiences, to be able to grasp the otherness of the phenomenon and not only what is already known about it (Gadamer, 2013).

Before the analysis began, all the interviews were read in their entirety to familiarize the researcher with the data. The analysis began with the researcher immersing herself in the interviews as a whole and identifying meanings as parts of the phenomenon. The meanings related to each other were grouped into clusters. At this preliminary stage of the analysis, the clusters were approached and questioned through reflections on how to uncover the essential meaning of the phenomenon. During the analysis, bridling allowed the process of understanding to slow down. While letting each meaning show itself against the background of the whole—and vice versa—a new understanding of the phenomena began to emerge. When all meanings had been identified, a new whole, the essential meaning, was revealed which contained a description of the phenomenon. When the essential meaning was uncovered and described, constituents were formulated. The constituents describe the variances in the essential meaning.

## Ethical considerations

The sensitive nature of this study warrants the consideration of genuine ethical issues. The ethical standards of the Helsinki Declaration (World Medical Association, 2013) were followed, and this study was approved by the Swedish Ethical Review Authority (Dnr 2023–02464–01).

When expressing interest in the study, the respondents used a university contact page to contact the researcher. After requesting more information, they consented to being contacted, and additional information was sent by the researcher. Participants were given opportunities to ask questions about the study and were informed that they could withdraw from the study at any time. A minimum of two weeks elapsed between the initial contact and the interview, ensuring that participants had sufficient time to thoroughly consider their decision to participate in the study.

To protect the participants’ identities and integrity, respect for and responsibility for confidentiality were taken. All participants were provided verbal and written information about the aim of the study, and informed consent for participation in this study was obtained by verbal consent that was recorded before the interviews started. Verbal consent was preferred since the sensitivity of gathered data, to keep the participants as anonyms as possible and a way to minimize the gathering of personal data. Recorded verbal consent is permitted according to the Swedish Ethical Board. All participants were offered support in contacting appropriate healthcare authorities after the interviews. The participants were given the opportunity to stop whenever they wanted during the interview, and the interviewer did not persuade them to continue if they showed any hesitation. Before the interview ended, all participants were given the opportunity to contact the interviewer if they felt a need to discuss any matters further or retract any statements made during the interview.

## Findings

The essential meaning of peer-perpetrated sexual violence, as described by young women, can be understood as living in an invaded existence, where an existential void emerges, and where the movement of life is disrupted.

“Invaded existence” means that the sexual violence becomes embedded in the body as an embodied memory, altering and limiting life. This memory is ever-present, leading to a state in which one’s entire being is reshaped and oriented around the sexual violence. Embodied memories evoke feelings of bodily loss, which is experienced as a deep sense of emptiness. The lostness and emptiness that follow sexual violence means that a part of the self has been taken away. These feelings permeate life, placing one’s existence under constant threat. Distancing oneself from the body becomes a means of regaining control while living in an invaded existence.

The bodily loss and the emptiness following sexual violence creates an existential void. The existential void manifests itself as a lost and empty existence filled with chaotic emotions in which one’s voice has been silenced. Life after sexual violence is redefined, with the past, present, and future irrevocably altered. This existential void manifests as a disruption in the movement of life, where existence is stalled and fractured by the invasion and ongoing threat of violence. The world becomes a dangerous place, and the fear of navigating life as a woman becomes a barrier to moving forward.

The essential meaning is further described by five constituents: *existing in a body constantly under attack, viewing the body as an object, fighting an inner battle—a struggle for survival, silence as armour*, and *navigating a dangerous world.*

## Existing in a body constantly under attack

Existing in a body constantly under attack means that the sexual violence continues to exist in the body, which is described through intrusive, vivid memories while awake and nightmares during sleep. The body can never fully rest and recover from the violence, because overwhelming memories of the sexual violence gradually emerge in fragments, bringing paralysing fear and confusion. This makes it challenging to be involved in daily tasks such as attending school or work, which, in turn, creates difficulties in managing daily life. Existing in a body constantly under attack also entails a sense of loss, both from the body and the self, described as if one no longer exists as a human being.

…it was as if someone had taken something from me…//…as if someone had stolen something…//…it’s something that happens to you in your soul…//…that you don’t exist…//…as if you’re not a human being. And it feels like the other person also feels and thinks that way… (8)

After experiencing sexual violence, a feeling arises that the other person has a claim to one’s body. Doubt arises about who the body belongs to after sexual violence, evoking an alienation that emerges when the body presents itself in a new way, feeling contaminated and used. The attacked body shifts from being familiar and known to being alien and unknown.

…I felt like my body disgusted me in some way. It felt like it didn’t belong to me… (12)

Intimacy and closeness are recognized as threats when existing in a body constantly under attack, as memories of the sexual violence are revived when intimacy and closeness are initiated.

…my body reacts, even if my mind does not…/…I can still experience panic attacks during intercourse. (1)

This entails that it becomes difficult, and sometimes impossible, to be intimate with someone else, even if there is a longing for closeness. Since the body under attack is seen as used, a feeling emerged that no one would choose what is depleted and broken, revealing a sense of being sentenced to a lifetime of abandonment and loneliness.

The relationship with one’s own body becomes complex, as the body is seen both as alien and as a cause of the sexual violence. A feeling of being unable to influence the situation, a sense of helplessness takes over, which means that the feeling of being stuck in the body can be so overwhelming that thoughts of wanting to end one’s life may arise.

…there was a lot of anxiety…/…and I think that’s also where my problems started…/…because there was a time when I attempted suicide because I didn’t know how to get out of it. (4)

Suicide attempts and thoughts of ending one’s life emerge from a desire to escape anxiety and feelings of helplessness, as well as being a cry for help. All energy is focused on managing life in the attacked body.

## Viewing the body as an object

Viewing the body as an object means that the body is perceived in a new way through the gaze of the other, where feelings of disgust, guilt, and shame dominate. After sexual violence, the body appears to be an object that must be managed and presented for control and protection. This means that an attempt to distance oneself from presence in the body is made as a way of handling the objectified body.

… that I was not just only a sex toy for men to use whenever they wanted, how they wanted … Accepting that I am enough as I am, has been difficult … much more difficult. (10)

When the body is perceived as dirty, contaminated, and used, it evokes feelings of disgust and revulsion, making distancing necessary to endure residing in the objectified body.

I showered because I felt like … dirty … //but it just didn’t work, I was just standing there still feeling absolutely disgusting. Like, you can’t wash it off, it’s stuck inside you somehow. (3)

The gaze of the other intensifies feelings of shame and vulnerability that arise from not being able to protect oneself. This also entails a sense of guilt for the sexual violence taking place, due to choosing to be in an exposed situation that made the sexual violence possible.

Because, in a way I had chosen to be with this guy, so I had, in some sense, chosen for it to happen. (3)

This can mean that others’ gazes towards the body are also perceived as threatening. One way to navigate life when viewing the body as an object is to hide the body and one’s femininity, creating protection against others’ threatening gazes.

…if I am very feminine or wear tight clothes…//…then I feel very, very small and vulnerable…//…so it has been very difficult because I haven’t been able to dress or look the way I want, as it feels like taking a risk. (11)

When experiencing the body as an object it reveals a sense of uselessness, futility, and disruption of movement in life. Attempts are made to reclaim the objectified body in order to survive and regain a feeling of existence. This is done through various activities such as keeping the body excessively clean, controlling food intake or involving oneself in self-harming sexual behaviours.

Ehm … and.I think that’s when I turned to the eating disorder and started controlling everything I ate. (4)

Presenting the objectified body as something chosen and desired by someone else decreases the feeling of the body as an object for the moment. This means that when an objectified body is given to another person, it involves a form of self-neglect that paradoxically evokes the feeling of being chosen.

…I might as well have sex in this way like that person wants…/…because then maybe that person will give me an acknowledgement, that I exist, that I am important, that I…that I mean something. (8)

For a brief moment, feeling seen confirms one’s existence in the world and is described as a way of feeling alive. Sexual risk-taking could also serve as a way of punishing the self and body, since the body is perceived as responsible for the prior sexual violence. Allowing others to invade the body it eventually evokes a sensation of perpetrating violence against oneself.

### Fighting an inner battle—A struggle for survival

“Fighting an inner battle” connotes a struggle for survival where one’s entire existence revolves around the feeling of being annihilated as a human being and existing in an existential void. Feelings of helplessness and hopelessness arise from the feeling of being unprotected and not being able to protect oneself. Helplessness is experienced as paralysing and frightening, revealing a feeling of being shattered as a human being.

I haven’t been able to protect myself…it makes you feels very powerless and small. (8)

When one’s own resistance is not visible to an individual, it illuminates an increased feeling of guilt and self-accusation. Fighting an inner battle inside the void also means dealing with the conflicting emotions that arise when active resistance was made during the sexual violence, but the physical resistance could not prevent or stop the ongoing violence.

…I hear myself saying no, no, no over and over again, and he doesn’t listen. It’s as if he doesn’t even register it…//…and eventually, I just go silent because it hurts more to lie there and hear myself say no while he doesn’t even acknowledge my words.//So, it felt like self-preservation to just…accept it. (11)

To be subjected to sexual violence can feel so unbearable that the only way to handle everyday life is to not acknowledge the experience and continue trying to live as one did before the sexual violence occurred. When distancing oneself from the violence, it does not feel as overwhelming and becomes a way to handle the chaotic emotions incurred by the sexual violence. When this is not possible, fighting an inner battle becomes a struggle to survive.

…I would say it has been a struggle for my life… (2)

When the experience of sexual violence does not align with one’s own perception of it, a discrepancy arises. The idea of what sexual violence entails can differ from how it manifests. This makes it difficult for individuals to navigate, and the feeling of fighting an inner battle intensifies. This inner battle could present itself as a depression, the origin of which they did not understand. This means that an individual can live for many years without recognizing the sexual violence as an act of violence and still be affected by it.

…didn’t really understand what happened…/…it was so unthinkable that it could happen.//…and I didn’t really know what to do…actually. so…*silent* So the whole situation was very…um…absurd for me…and. I couldn’t really understand what it was until maybe a year later. (8)

First, when the extent of the sexual violence becomes clear, an understanding of what the individual has experienced emerges. This awakening evokes feelings of pain, anxiety, anger, and sorrow, and can sometimes manifest as various forms of self-destructive behaviour as a way to handle the resulting inner chaos.

## Silence as armour

“Silence as armour” means that silence becomes both a protection and a barrier against the outside world after sexual violence. As an armour, silence protects against revealing or exposing vulnerabilities. Staying silent can offer a shield, against reactions of the outside world in an existential void. This means that silence as an armour can create a safe space for reflection, allowing one to gather one’s thoughts and emotions and by serving as a form of self-defence. At the same time, silence as an armour can also create a barrier, as by blocking communication and creating emotional distance, silence disrupts the movement of life.

…during that period, I had a very hard time. I isolated myself a lot from my friends…/…and I was just alone in my room, not talking to my family…/…I just wanted to be alone and didn’t want to participate in activities, and I had a lot of difficulty with closeness.//…I think that’s why I became so isolated, because it felt like it drained my energy… (3)

When silence is used as an avoidance mechanism, it builds a distance between friends and family, preventing an understanding and deeper connections. This means that silence as armour can be protective, but at the same time functions as an obstacle, creating more distance between people and relationships. Silence as a barrier also means an inability to put what has happened into words. Feelings of physical and emotional isolation arise when not having anyone to share the experience with. This isolation can serve as both protection and a barrier against the outside world.

…it was easier when no one knew because then it was only on me. When everything comes out, you have to think about what they will think? (3)

Distancing creates a lack of understanding among others, and the deep connection that defines close relationships with friends and family is lost, which in turn creates feelings of loneliness. Loneliness feels profound because all relationships in life, as they were known before, change after sexual violence, as silence creates distance in those relationships. Fear and a growing concern arise that family and friends will not understand. This evokes fear that it becomes necessary to handle and manage other people’s reactions and emotions after the revealing.

…but I felt like I needed to take responsibility for their feelings in some way…yes…to bear it myself. I didn’t want to hurt them in any way, by telling them that I had been through this. (10)

Silence is also a way to protect and take responsibility for one’s parents, siblings, and friends by not telling them about the sexual violence. It was described as sparing, for example, one’s parents’ worry and emotional distress of having a child who has been subjected to sexual violence. However, when adults chose silence or chose not to see the abuse, silence became a barrier. It was experienced as a betrayal by the adult world and could lead to further victimization.

I mean it is really heartbreaking to realise that there were people [adults] who could have prevented it..but they didn’t. (11)

If attempts are made to disclose the sexual violence that is then met with scepticism or denial of the experience, it is perceived as being subjected to violence again, creating emotions of further victimization. By not acknowledging the act of violence, legitimization of the sexual violence occurs, and a fear of not being believed arises. This means that the responsibility for sexual violence remains with the individual, further intensifying feelings of shame and guilt, and the trust and ability of others to receive and understand the experience decreases.

…then she said, ‘Well, you should have just said no to him, pushed him away or something.’//I felt very dumb.//…why am I walking around with so much anxiety, I shouldn’t really have anxiety.//…it felt like it was partly my fault, but also that it was my fault for feeling bad about it. (8)

When an individual shares the experience of sexual violence and is then met with distrust or blame, it reinforces the feeling of being shattered and the idea that the violent act was deserved. Distrust from others strengthens one’s reliance on silence as a protection and makes it increasingly difficult to break. This can lead to a diminishment of one’s own experience of the sexual violence and a deeper sense of loneliness arises.

## Navigating a dangerous world

*Navigating a dangerous world* implies that after the sexual violence, the world is experienced as a dangerous and unpredictable place. Disruption of the movement of life emerges as the fear of being subjected to sexual violence again takes hold and evokes feelings of not feeling safe anywhere. Navigating a dangerous world means that fear and insecurity limit one’s freedom in life and become an obstacle in living life to the fullest. The lack of freedom, when fear takes over and safety disappears, creates difficulties in living life as desired.

…I see violence precisely everywhere in society…/ … I am never protected from it because there are violent men everywhere. I feel that it limits me a lot in life… (2)

Navigating a dangerous world involves constantly adapting to an unpredictable environment to feel safe. This entails an awareness of how one’s own body interacts with other bodies in the world, where the fear of being subjected to further sexual assault leads to active avoidance of certain places and people. Navigating a dangerous world also entails navigating dangerous men. The fear of being in a relationship with men or unexpectedly being alone with men is described as potentially dangerous and limiting life. A feeling of being unprotected in the world arises and means that navigating a dangerous world makes it difficult or impossible to return to life, as was known before. When the extent of the sexual violence becomes evident, it affects one’s entire existence, revealing a disrupted life movement.

…you kind of just go around, not really living your life. Instead, you just go around, trying to survive. (8)

The young women continue to navigate in a dangerous world long after the initial act of violence has occurred. Awareness of vulnerability to violence becomes even more pronounced during pregnancy and when becoming a parent. Pregnancy can result in feelings of being in an unsafe situation in which control is lost.

… during the birth of my first daughter … //…it was a rather brusque midwife who came in for the vaginal examination and she didn’t ask for permission, and I just completely lost it. (9)

Fear and avoidance may arise when thinking about the childbirth and dependency on unfamiliar caregivers. Feelings of being re-victimized during pregnancy and birth emerged when trying to protect their bodies from the hands of unknown people. Unwanted closeness wakes embodied memories to live, and birth can be experienced as traumatic when overwhelming feelings of not being in control emerge. However, pregnancy and birth can also be experienced as empowering and healing when the strength of the body becomes visible.

It is important to feel that I own my body and that I am in charge…//and it helped me in a way… the birth…// … it became a really good thing for my self-confidence and I gained a new belief in my body. (11)

A sorrow emerged from the realization that life will never be the same as it was before the sexual violence, yet as everyday life continues, the world remains a dangerous place, and the view of oneself is forever changed. Navigating a dangerous world means becoming aware of one’s vulnerability to violence.

What has impacted me the most about the abuse is that I haven’t felt safe anywhere since. (1)

It is described by the young women that experiences of sexual violence shape the direction of the rest of their lives. When school is affected, new directions may need to be taken to complete one’s education, leading to sorrow, disrupted movement in life, and a sense of loss. Navigating a dangerous world can also entail making new choices for the future, as certain opportunities may have been taken away after the sexual violence.

## Methodological reflections

Reflective Lifeworld Research has a well-established ontological and epistemological foundation that gives the method a strong scientific foundation to stand upon (Dahlberg et al., 2008). To maintain objectivity, the methodological principles of openness, bridling, and a reflective attitude towards the phenomena were kept at the forefront during the entire research process (Dahlberg et al., 2008; van Wijngaarden et al., 2017). Uncontrolled preunderstanding can be a threat to validity in lifeworld studies, since it can complicate the understanding of the phenomena (Palmér et al., 2022). In this study, one way of handling preunderstanding was through bridling, which included critical reflection within the research group and discussions during seminars with senior researchers. In a phenomenological lifeworld research study, validity is associated with describing the meanings of a phenomenon (Dahlberg et al., 2008). In this study, lifeworld interviews were conducted to obtain meaningful descriptions of the phenomenon. The interviews in were conducted through online meetings or telephone. The purpose was to give the participants the opportunity to participate regardless of where they lived. When using lifeworld interviews, the interviewer needs to get close to the participant to deepen their reflection on the phenomenon and to be able to gather in-depth data a sense of immediacy is required between the interviewer and the participant (Dahlberg et al., 2008). The immediacy of online meetings and phone interviews can be discussed since immediacy relies on interpersonal connection (Dahlberg et al., 2008). When using online meetings or telephone calls as methods for gathering data, a sense of proximity can be difficult to achieve, which may affect the richness of the data. It is possible that in-person interviews may have allowed for a deeper engagement with the phenomenon, potentially influencing the overall outcome of the interviews. Some reflections might have become more intense in their exchange during an in-person interview. However, given the results, the interviews provided both depth and variations of the phenomenon. According to Thunberg and Arnell (2022) showed that there seems to be little or no difference between online and in-person interviews, as long as the interviews are well-planned, the audio and visual software works, and the internet connection is stable. For this study, online meetings were perceived as less intrusive than in person meetings, which seemed to give the young women a feeling of safety during the interview, which could be positive for the richness and variation of the gathered data.

Considering that young people today are familiar and accustomed to using different digital methods for communication, this method of interviewing might be preferred. According to Thunberg and Arnell (2022), when conducting research with vulnerable groups, sensitive topics or people who have difficulties participating because of distance, online meetings are experienced as positive. To achieve validity, the researcher strives for rich descriptions by asking open and reflective questions and allowing the phenomena to guide the interview, as requested by van Wijngaarden et al. (2017). The choice to hold online or personal communication was pragmatic, but as a result it went out well and the interviews contained rich descriptions of the meanings.

In terms of validity, the young women included in this study were homogeneous. They were all ethnic Swedes with Swedish as their native language. Information about the study was only given in Swedish and was only available on websites directed to young people in Sweden. This excluded other possible participants, and possibly also an opportunity to deepen the understanding of the phenomenon. The inclusion of young women from different countries and diverse ethnic backgrounds might have resulted in a variation in meanings. The findings of this study are presented as an essential meaning with its constituents, which is a strength in terms of generalizations of the result. However, phenomenological findings should always be understood in their context (Dahlberg et al., 2008; van Wijngaarden et al., 2017). In this study, the context of Sweden and Swedish-speaking young women should be considered. The results are transferable to similar contexts and groups.

## Discussion

The findings will further be developed in the discussion with the support of existential philosophy, caring science, and empirical research to deepen the understanding of young women’s experiences of peer-perpetrated sexual violence. The results of this study revealed that young women’s lives were significantly shaped, altered, and impacted by sexual violence. These results support previous studies and are consistent with existing literature in the field (Basile et al., 2020; dos Reis et al., 2017; Stockman et al., 2023, 2024; Tarzia et al., 2024).

However, in this study the findings go deeper and reveal how peer-perpetrated sexual violence is experienced by young women on an existential level and was described through the essence of the phenomenon. The invasion of the existence that emerges after sexual violence where the existential void arises is so overwhelming that everything else in life is put on hold, their voices are silenced, and the young women are left alone to fight for survival. The inner chaos inside the existential void threw them into isolation, as they struggled to understand what had happened.

When the sexual violence became embedded in the body as an embodied memory, it started to limit life. The traumatic memories from the past are constantly present and reveal themselves through flashbacks, anxiety, and nightmares. Therefore, when memories from the past become embodied, they continue to exist in the present. This can further be understood, as the phenomenological philosopher Merleau-Ponty (1999) describes, as the lived body which is an inseparable unity of the body and soul. The lived body gives humans access to the world, since we know the world through our body. In this way, bodily involvement makes the world more meaningful. This implies that every change in the body leads to a change in one’s ability to engage with the world. Any change in the body alters access to the world and life itself. Merleau-Ponty (1999) argues that a person not only has a body, but she *is* her body. Because the body is constantly present, there is no opportunity to escape from it. This becomes visible when the feeling of being objectified after the sexual violence is internalized and participants move from being an active participant in life to experiencing a disruption in the movement of life. This finding aligns with previous research where adult women described the experience of sexual violence as something that disrupted their lives and forced them into a constant presence of fear and mistrust (dos Reis et al., 2017).

As Merleau-Ponty (1999) notes, being disrupted from the body that gives humans access to the world distances the person from the world, and the meaning of life is changed as the bodily involvement in it changes. A consequence of being subjected to peer-perpetrated sexual violence is that young women began to distance themselves from their own bodies. They struggled to view themselves as women who have the ability to engage in the world as free subjects without being assessed, since femininity and the female body were related to the threat of being subjected to sexual violence. Based on these empirical results, Marion Young’s (2005) philosophy highlights that women primarily experience their bodies through how they are viewed and assessed by others. This leads to a sense of alienation from the body, and since the world is experienced through the body, the self also feels alienated within the world. Cahill (2000) describes how women restrict their movement in the world for safety, since the threat against the female body is almost always specifically sexualized, and how women’s bodily behaviour and being in the world are shaped by the constant presence of the threat of sexual violence. This may explain the lived experiences observed in the present study. However, as Young (2005) states, embodiment and accessibility in the world are not the same for men and women. Women are convinced to relate to their bodies as objects, and the embodiment and bodily relationship with the world influences how women experience the world (Young, 2005). Beauvoir (2002) described how passivity arises when a young woman sees herself as an object. In this existence, she has lost confidence in her own abilities and has gained mistrust of her own body and of the world. Since the body is our access to the world, it becomes, for the young woman with experience of peer-perpetrated sexual violence, something that separates her from the world rather than connecting her to it (Beauvoir, 2002).

According to previous research on young adults being subjected to violence in their first sexual experiences, questions have been raised about what is internalized as healthy sexual behaviour and what is not (Överlien et al., 2020). Studies has shown that being subjected to sexual violence is associated with unhealthy sexual behaviours among adolescents (Basile et al., 2020). Also, individuals with a history of sexual violence tend to engage in higher-risk sexual behaviour (Stockman et al., 2023, 2024). This was also identified in the findings of this study. Historically, Beauvoir (2002) stated that a part of femininity is involved in pleasing and being an object for others. This means that a girl is forced early on to relate to the image of herself, how she appears and looks to others. It is the outside world that defines her existence, not her own being in the world (Beauvoir, 2002). After peer-perpetrated sexual violence, the young women became painfully aware that they no longer could define their own existence, since being subjected to sexual violence now defines who they are.

The results indicate that young women subjected to peer-perpetrated sexual violence experience their existence as lost and empty, where the movement of life is disrupted. The sense of loss and emptiness that follows peer-perpetrated sexual violence states that a part of the self has been taken away. Tarzia et al. (2024) found in their study how women felt something has been stolen from them and that their identity was changed after experiencing sexual violence. The sense of loss and emptiness seems to be a finding that is associated with experiences of sexual violence regardless of time and place. Self-hate and self-destructive behaviour were described as one way of handling these chaotic feelings, when life suddenly turns into a battle for living. Life is now different than it was before the peer-perpetrated sexual violence occurred, and the surrounding world becomes a dangerous place. Similar findings, described by Braennstroem et al. (2020), were that young women subjected to sexual violence withdrew from social and public environments, as they felt unsafe. It becomes difficult for the young women to continue everyday life, as they feel that their existence is under constant threat. They have this imminent feeling of being subjected to violence again, and the young women begin distancing themselves from the world to protect themselves, which leads to further isolation and loneliness. Fear is a common finding in sexual violence research and is often described as fear of being assaulted again, fear of leaving home and fear of other people (dos Reis et al., 2017; Stockman et al., 2023; Tarzia et al., 2024). When the young women distanced oneself from the situation and the body it made life more manageable, and it became a way of handling the invasion of existence due to peer-perpetrated sexual violence.

As shown in the findings of this study, the movement of life is disrupted after being subjected to peer-perpetrated sexual violence and for the young women this is a fundamental existential experience that alters life and according to Galvin and Todres (2011) the absent of well-being is felt in the body as suffering. Through the existential view of humans, suffering needs to be understood as being more complex than illness and well-being, affecting an individual more deeply than simple physical health (Galvin & Todres, 2011). The relationship between existence and caring can be used as a foundation for understanding complex phenomena, such as young women’s lived experiences of peer-perpetrated sexual violence. Todres et al. (2014) describe a way of trying to understand existential concerns and suffering by trying to reach others’ *insiderness*. Reaching others’ *insiderness* can be achieved by a lifeworld perspective staying close to the embodied experience, which can help healthcare professionals support women in finding a way to move forward (Todres et al., 2014). Galvin and Todres (2011) identified mobility as an important aspect of being able to feel well-being. Mobility describes access to existential possibilities of moving forward, and this can be felt in life as a way of being able to live life as one wishes. One way of supporting young women in moving towards well-being is to help them restore a sense of being at home in one’s body again, and the feeling of coming home can be described as a place where acceptance, rootedness, and peace emerge (Galvin & Todres, 2011).

Earlier studies have shown that healthcare professionals need support to increase their trust and confidence when encountering patients with a history of being subjected to violence (Tarzia et al., 2021) but they also need support to endure stories told when encountering young women with experiences of peer-perpetrated sexual violence. If caring cannot ease suffering, and healthcare professionals are not able to endure the young women’s stories, this might reinforce objectification, passivity, and further victimization. Having a lifeworld perspective, in which existential caring and awareness can take place, might support health care professionals to come closer to the lived experiences (Todres et al., 2014).

The conclusion of the study is that peer-perpetrated sexual violence creates deep existential wounds that change the young women’s view of themselves and reshape their entire lives. For healthcare professionals, recognizing the importance of existential awareness is essential to effectively support young women with a history of peer-perpetrated sexual violence, as living with embodied memories that are always present requires learning to live life in a new way.

## Relevance to clinical practice

Peer-perpetrated sexual violence affects young women’s lives by creating an existential void where they are silenced and left alone navigating the inner chaos that follows. When healthcare professionals encounter young women with a history of sexual violence, they need to have knowledge on how profoundly young women’s lives are affected. If healthcare professionals have the capacity to approach lived experiences of peer-perpetrated sexual violence, existential awareness can help them to create safe environments where silence and loneliness can be broken, and the life movement can restart again. It is crucial to develop care that recognizes sexual violence as an existential issue, since embodied memories never leave, and therefore reshape the lives of young women.

How deeply sexual violence changes young women’s views of themselves and the world around them is important not only when healthcare professionals encounter young women, but also when developing clinical practice and education for healthcare professionals. This can be achieved through a lifeworld perspective and by existential awareness that entails a deeper understanding of the individual’s needs. If healthcare professionals cannot meet young women’s disclosure about their experiences of peer-perpetrated sexual violence with adequate knowledge and skills, the present study indicates that there is a risk that they will withdraw, and their silence and suffering will continue. Knowledge and insights from this article are important for contributing to the development of caring for this very vulnerable group. Future research should include knowledge of how young women recover from peer-perpetrated sexual violence and how healthcare professionals can develop their caring practice to adopt a more existential approach when encountering patients with a history of sexual violence.

## Data Availability

Data material is stored at the University of Borås and may be made available upon reasonable request after ethical consideration.

## Notes

JI participated in the planning of the study, collected data, analysed the material, and lead the writing of the manuscript. LP and ML participated in planning and, analysis of the material, and contributed to writing and revising the manuscript. All authors have read and approved the final manuscript.
